# The role and mechanism of biological collagen membranes in repairing cartilage injury through the p38MAPK signaling pathway

**DOI:** 10.1186/s13018-023-04261-y

**Published:** 2023-11-06

**Authors:** Li-Bo Yuan, Tao Jin, Ling Yao, De-Hong Yin, Yong-Qing Xu

**Affiliations:** Department of Orthopedics, The 920 Hospital of Joint Logistics Support Force of the Chinese People’s Liberation Army, Kunming, China

**Keywords:** Joint cartilage defect, P38MAPK signaling pathway, Biological collagen membrane, Rabbit

## Abstract

**Objective:**

To explore the mechanism of the p38MAPK signaling pathway in repairing articular cartilage defects with biological collagen membranes.

**Methods:**

Thirty-two healthy adult male rabbits were randomly divided into a control group (*n* = 8), model group (*n* = 8), treatment group (*n* = 8) and positive drug group (*n* = 8). The control group was fed normally, and the models of bilateral knee joint femoral cartilage defects were established in the other three groups. The knee cartilage defects in the model group were not treated, the biological collagen membrane was implanted in the treatment group, and glucosamine hydrochloride was intragastrically administered in the positive drug group. Twelve weeks after the operation, the repair of cartilage defects was evaluated by histological observation (HE staining and Masson staining), the degree of cartilage repair was quantitatively evaluated by the Mankin scoring system, the mRNA expression levels of p38MAPK, MMP1 and MMP13 were detected by real-time fluorescence quantitative PCR (qRT-PCR), and the protein expression levels of p38MAPK, p-p38MAPK, MMP1 and MMP13 were detected by Western blotting. The results after the construction of cartilage defects, histological staining showed that the articular cartilage wound was covered by a large capillary network, the cartilage tissue defect was serious, and a small amount of collagen fibers were formed around the wound, indicating the formation of a small amount of new bone tissue. In the treatment group and the positive drug group, the staining of cartilage matrix was uneven, the cytoplasmic staining was lighter, the chondrocytes became hypertrophic as a whole, the chondrocytes cloned and proliferated, some areas were nest-shaped, the cells were arranged disorderly, the density was uneven, and the nucleus was stained deeply. The Mankin score of the model group was significantly higher than that of the control group, while the Mankin scores of the treatment group and positive drug group were significantly lower than that of the model group. The results of qRT-PCR detection showed that compared with the control group, the expression level of the p38MAPK gene in the model group did not increase significantly, but the gene expression levels of MMP1 and MMP13 in the model group increased significantly, while the gene expression levels of MMP1 and MMP13 decreased significantly in the treatment group and positive drug group compared with the model group. The results of Western blot detection showed that compared with the control group, the expression level of p38MAPK protein in the model group was not significantly increased, but the phosphorylation level of p38MAPK protein and the protein expression levels of MMP1 and MMP13 were significantly increased in the model group, while the phosphorylation level of p38MAPK protein and the protein expression levels of MMP1 and MMP13 in the treatment group and positive drug group were significantly lower than those in the model group.

**Conclusion:**

The biological collagen membrane can regulate the expression of MMP1 and MMP13 and repair the activity of chondrocytes by reducing the phosphorylation level of p38MAPK and inhibiting the activation of the p38MAPK signaling pathway, thus improving the repair effect of articular cartilage defects in rabbits. The P38MAPK signaling pathway is expected to become an important molecular target for the clinical treatment of cartilage defects in the future.

## Introduction

Articular cartilage is the most common type of cartilage in the human body, which can reduce the stress of joints, reduce the friction between knee joints, increase buffering and absorption force, and has a protective effect. Cartilage injury can cause joint dysfunction, resulting in long-term pain and discomfort [1]. Articular cartilage belongs to hyaline cartilage, and the matrix is collagen type II expression, including chondrocytes and nonvascular extracellular matrix (ECM). It has four layers: superficial, middle, deep and calcified cartilage [2]. Chondrocytes exist in the ECM of the surface and deep regions. Morphologically, primary fibrosis occurs from the superficial zone of articular cartilage, followed by cracks in the middle and deep layers, and finally calcified cartilage disappears, exposing subchondral bone to the articular cavity and leading to bony ivory [3–5], resulting in cartilage destruction and finally osteoarthritis (OA). ECM degradation is the most important factor in cartilage destruction, while mechanical stress, inflammatory factors and X-rays in articular cartilage can induce ECM degradation or loss, activate the p38MAPK signaling pathway, induce chondrocyte apoptosis, and finally cause cartilage injury [6].

Matrix metalloproteinases (MMPs) are divided into collagenase, gelatinase, matrix lysin and matrix lysin according to substrate specificity [7]. MMPs are important indicators for the detection of inflammation and cartilage destruction. MMPs can degrade the extracellular matrix composed of proteoglycan and collagen and destroy cartilage. In OA cartilage, the expression of MMP-1, MMP-3, MMP-13 and MMP-28 is stronger [8], and MMP-13 can directly degrade ECM and destroy cartilage [9].

The P38MAPK signaling pathway not only can transmit extracellular signals into cells but also is the key pathway of cartilage destruction in knee osteoarthritis and can regulate a variety of physiological and pathological processes, such as cell inflammation and cell differentiation [10]. The P38MAPK signaling pathway can regulate the secretion of MMPs by chondrocytes, including MMP-1 and MMP-13 [11], and participate in a variety of biological processes: (1) acting on type II collagen, destroying cartilage and causing chondrocyte hypertrophy; (2) upregulating IL-1 and IL-6, regulating cell growth and differentiation; and (3) promoting the synthesis of cyclooxygenase, destroying cartilage and inducing joint pain. (4) Chondrocytes differentiate into fibroblasts and hinder cartilage regeneration [12].

At present, collagen as scaffolds for cartilage tissue engineering is mainly type I and type II collagen, which is beneficial to the adhesion, proliferation and differentiation of chondrocytes [13]. It has been found that collagen membranes have good biocompatibility, act as mechanical barriers and provide space protection [14–17]. Therefore, in this study, a pathological method was used to study the effect of biological collagen membranes on the repair of articular cartilage defects in rabbits, and its molecular mechanism was deeply studied by molecular biology methods. This study provides experimental data and a theoretical basis for further elucidating the molecular mechanism of articular cartilage defect repair with biological collagen membranes.

## Materials and methods

### Animals

A total of 32 healthy adult male rabbits (1.5–2.0 kg) were purchased from the Animal Experimental Center of Kunming Medical University. They were qualified in animal quarantine and were II in clean grade. Adequate food and tap water were freely available to all animals, who were observed for 1 week before random experimentation. All experimental personnel conducted experiments under the supervision and guidance of the Laboratory Animal Ethics Committee of The 920 Hospital of Joint Logistics Support Force of the Chinese People’s Liberation Army, and the approval number of our experiments is 2023-073-01.

### Modeling and grouping treatment

Eight rabbits were randomly selected as the normal group. The other 24 rabbits were subjected to the modeling treatment. After anesthesia with pentobarbital sodium (30 mg/kg) injected into the auricular vein, the limbs were placed in a supine position, the skin was prepared and disinfected near the right knee, the patella was tested from the inside of the patella, the articular surface of the femoral condyle was exposed, a defect with a diameter of 3 mm deep to the subchondral bone was drilled into the cartilage surface, and bone fragments and blood were removed from the injured site. After successful modeling, the model rats were divided into a model group, a biological collagen membrane treatment group (BCM group) and a positive drug group (GH group).

In the BCM group, a biological collagen membrane was implanted at the defect site, and the implantation amount was determined to fill the defect. On the next day after the positive drug formation model was completed, glucosamine hydrochloride was given by intragastric administration (150 mg/kg/d). After the operation, rabbits were kept in a single cage without a fixed knee joint. Twelve weeks after the operation, rabbits in each group were killed by air embolization of the auricular vein, and knee joint samples were obtained through the original incision.

### Histological observation of cartilage

The distal part of the femur of the knee joint was fixed in 4% paraformaldehyde solution for 48 h, decalcified in 10% EDTA solution for 2 weeks, dehydrated and embedded, and 6-μm-thick sections were cut. The repair of cartilage defects was observed under a light microscope after routine HE and Masson staining.

### Mankin score

After staining, the cartilage of each group was scored according to the Mankin scoring system under a light microscope. Mankin score system [18]: tissue structure: normal (0), irregular surface (1), pannus formation (2), loss of cartilage surface (3); number of cells: normal (0), high cellular (1), cluster (2), low cellular (3); tidal line: intact (0), blurred (1), slight fracture (2), fracture (3). Staining was scored as follows: normal/slightly decreased (0 points), decreased reflex layer staining (1 point), decreased interregional matrix staining (3 points), and no interregional matrix staining (4 points). According to the score of 0–13, the higher the score, the more serious the articular cartilage injury.

### Real-time fluorescence quantitative PCR

Total RNA was extracted from articular cartilage by adding TriQuick Reagent (Solarbio, Beijing, China), chloroform, isopropanol and 75% ethanol. Total RNA was reverse-transcribed to cDNA with a Servicebio ®RT First Strand cDNA Synthesis Kit (Servicebio, Wuhan, China). Subsequently, qPCR was performed using 2 × SYBR Green qPCR Master Mix (High ROX) (Servicebio, Wuhan, China) on a StepOnePlus real-time PCR system (Applied Biosystems, Thermo Fisher Scientific, Inc.). GAPDH was used as a standardized control. The sequences of the primers (Shanghai Jierui Biological Engineering Co., Ltd.) are shown in Table 1.

**Table 1:** Primer sequence information

Gene name	Forward primer (5′ → 3′)	Reverse primer (5′ → 3′)
P38MAPK	AGAAGTCAGAGTTCAGAGGCGTCC	AGTAGAAGGCTGTCACCAAGCCAAC
MMP1	CCTGATGTGGCTCAGTTCGT	GTCCACATCTGCCCTTGACA
MMP13	TGAGCTGGACTCATTGCTGG	AGACTGCATTTCTCGGAGCC
GAPDH	GTATGATTCCACCCACGGCA	CCAGCATCACCCCACTTGAT

### Western blot

Articular cartilage (0.1 g) was weighed and lysed for 30 min on ice with RIPA lysis buffer containing PMSF (R0010). The supernatant was obtained by centrifugation for 20 min at 4 ℃ and 12,000 rpm, that is, the total protein. The protein concentration was measured by a BCA protein assay kit (PC0020, Solarbio). Equal amounts of protein samples were separated by SDS‒PAGE and then transferred to PVDF membranes (IPVH00010, Millipore). The membrane was blocked with 5% skim milk at room temperature for 1 h and then incubated with antibodies against p38MAPK (AF6456, Affinity), p-p38MAPK (AF4001, Affinity), MMP1 (AF0209, Affinity), MMP13 (AF5355, Affinity) and beta actin (AF7018, Affinity) overnight at 4 °C. Then, the membrane was incubated with the secondary antibody at room temperature for 2 h. The protein bands were detected by ECL reagent (WBKLS0100, Millipore) and observed by a Bio-Rad chemiluminescence imaging system (Chemidoc XRS+, Bio-Rad). ImageJ software was used to quantify the scanned image.

### Statistical analysis

Statistical analysis was performed using SPSS software (V20.0, IBM, Chicago, USA). Data are presented as the mean ± SD. Groups were compared using one-way ANOVA. When the variance was homogeneous, the S–N–K (Student–Newman–Keuls) method was used to compare the two groups; otherwise, Tamhane’s T2 method was used when the variance was uneven. A *p* value of < 0.05 was considered to be statistically significant.

## Results

### Observation of cartilage histomorphology

**Fig. 1:**
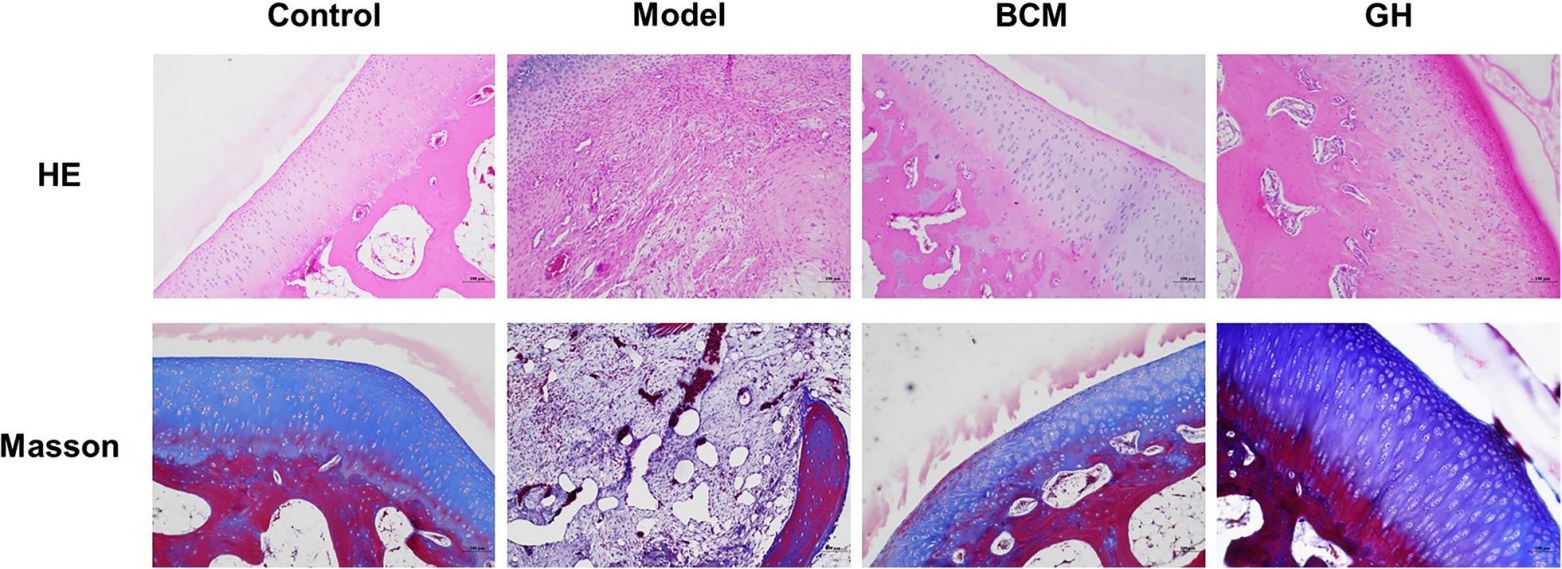
HE staining of articular cartilage(magnification, ×100) and Masson staining of articular cartilage (magnification, ×100)

To evaluate the defect and repair of articular cartilage, HE staining and Masson staining were performed on articular cartilage in each group. The results in Fig. 1 show that in the control group, the structure of articular cartilage was normal, the cells were arranged regularly, there were no cracks, the articular cartilage was divided into four layers, the surface chondrocytes were parallel to the articular surface, and the chondrocytes were distributed in a slender shape in the lacunae; the collagen fibers of the transitional layer chondrocytes were parallel to the surface articular surface and gradually turned oblique. The base of the radiation layer is characterized by the perpendicular and columnar arrangement of chondrocytes to the surface of the cartilage, the tidal line can be seen, and the hypertrophy of chondrocytes in the calcified layer is round or oval, as shown in Fig. 1. In the model group, the wound was covered by a large capillary network, the cartilage tissue defect was serious, and a small amount of collagen fibers were formed around the wound, indicating the formation of a small amount of new bone tissue. In the BCM group and the GH group, the staining of cartilage matrix was uneven, the cytoplasmic staining was lighter, the chondrocytes became hypertrophic as a whole, the chondrocytes cloned and proliferated, some areas were nest-shaped, the cells were arranged disorderly, the density was uneven, and the nucleus was stained deeply.

### Mankin rating results

**Fig. 2:**
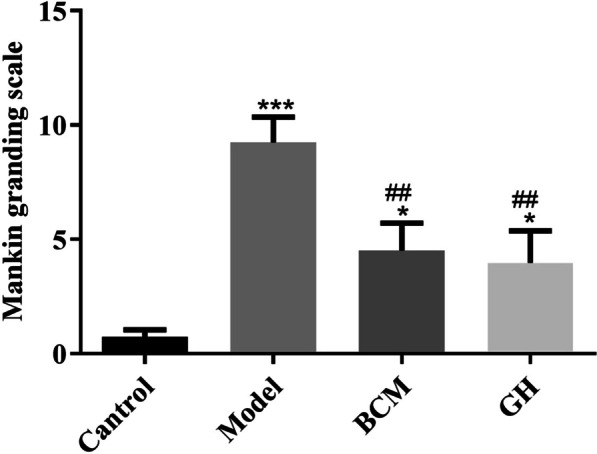
Mankin scoring results (*p < 0.05, ***p < 0.001 vs. control group; group, ^##^*p* < 0.01 vs. model group)

To quantitatively evaluate the defect and repair of articular cartilage, the articular cartilage of each group was scored by Mankin. As shown in Fig. 2, the score of the model group was significantly higher than that of the control group (*p* < 0.001). The scores of the BCM group and the GH group were significantly lower than those of the model group (*p* < 0.01).

### The mRNA expression levels of p38MAPK, MMP1 and MMP13

**Fig. 3:**
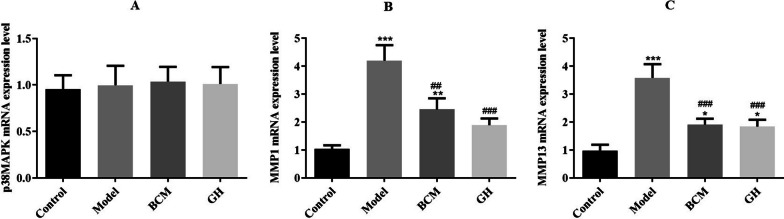
qRT-PCR detection of gene expression levels of p38MAPK (**A**), MMP1 (**B**), and MMP13 (**C**) in articular cartilage (*p < 0.05, ***p < 0.001 vs. control group; ^##^*p* < 0.01, ^###^*p* < 0.001 vs. model group)

To understand the effect of the expression levels of p38MAPK, MMP1 and MMP13 in articular cartilage on the defect and repair of articular cartilage at the gene level. The expression level of each gene in articular cartilage was measured by qRT-PCR. As shown in Fig. 3, there was no significant difference in the expression level of p38MAPK mRNA in cartilage tissues of each group. The expression levels of MMP1 and MMP13 mRNA in the model group were significantly higher than those in the control group (*p* < 0.001). The expression levels of the MMP1 and MMP13 genes in the BCM and GH groups were significantly lower than those in the model group (*p* < 0.01).

### The protein expression levels of p38MAPK, p-p38MAPK, MMP1 and MMP13

**Fig. 4:**
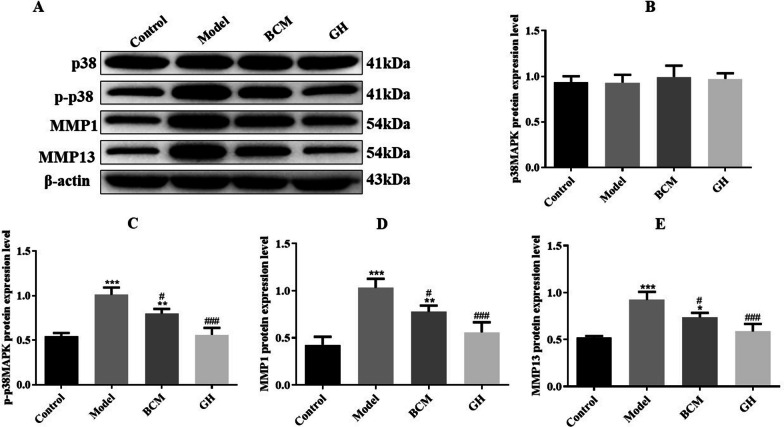
Western Blot detection of protein expression levels of p38MAPK, p-p38MAPK, MMP1, and MMP13 in articular cartilage (**A**) WB original strip (**B**–**E**) WB analysis bar chart (*p < 0.05, **p < 0.01, ***p < 0.001 vs. control group; ^#^*p* < 0.05, ^###^*p* < 0.001 vs. model group)

The protein expression levels of p38MAPK, p-p38MAPK, MMP1 and MMP13 in articular cartilage were analyzed by Western blotting. As shown in Fig. 4, there was no significant difference in the expression of p38MAPK protein among the three groups. Compared with the control group, the protein expression levels of p-p38MAPK, MMP1 and MMP13 in the model group were significantly increased (*p* < 0.001), while the protein expression levels of p-p38MAPK, MMP1 and MMP13 in the BCM group and GH group were significantly lower than those in the model group (*p* < 0.001).

## Discussion

An attractive alternative to cartilage repair is tissue engineering, whose testing and optimization has yielded many promising cartilage grafts and complex bioreactor systems for in vitro graft culture over the past 2 decades [18–23]. Modern tissue engineering includes three basic elements: cells (chondrocytes, stem cells or progenitor cells), biodegradable scaffolds and growth factors. This method is particularly suitable for the repair of articular cartilage [24] because the scaffold provides a three-dimensional network for the growth of chondrocytes [25] and serves as a medium for cell signal transduction and interaction. However, the physical and biochemical properties of these stents are crucial [26]. In this study, it was found that biofilms can effectively improve the pathological condition of cartilage defect and promote the repair of cartilage defects, which is consistent with the results of previous studies.

In the progression of osteoarthritis, it begins to damage the surface of the articular cartilage and then spreads to the subchondral bone, leaving fibrillation and ulcers. It is generally believed that the cartilage matrix (mainly including proteoglycan and type II collagen) undergoes structural changes through the specific role of matrix metalloproteinase family members. MMP1 and MMP13 are two kinds of collagen hydrolases that have been proven to play a major role in changes in collagen [27]. At present, many studies at home and abroad have proven that IHH and RUNX2 lead to bone and joint hypertrophy through excessive maturation and differentiation of chondrocytes and further upregulate MMP protein, thus promoting the progression of osteoarthritis [28, 29].After cartilage injury, pro-inflammatory cytokines including IL-1β, IL-6, tumor necrosis factor-α (TNF-α), matrix metalloproteinase-1 (MMP-1), MMP-13 and other decomposing factors increase rapidly, causing inflammation, chondrocyte apoptosis and cartilage ECM degradation [30]. Our results showed that the expression of MMP1 and MMP13 genes and proteins decreased significantly after biofilm treatment, suggesting that biofilms can promote cartilage repair by inhibiting MMP, which is consistent with previously reported studies.

Cartilage matrix and chondrocytes together constitute articular cartilage, and the function of articular cartilage is mainly maintained by normal metabolism of chondrocytes [31]. Therefore, excessive apoptosis of chondrocytes may be an important factor leading to cartilage injury. Silk crack the original activated protein kinase (Mitogen-activated protein kinases, MAPKs) signal transduction pathway of chondrocytes apoptosis is very important. The degeneration of articular cartilage and osteoarthritis are closely related to MAPKs signaling pathway [32]. As an important branch of MAPK signal, the p38 pathway is critical for the growth, apoptosis, and inflammatory reactions of chondrocytes. Through inhibiting p38 pathway, not only the apoptosis of chondrocytes was inhibited, and the further cartilage recovery was promoted but also the generation of inflammatory factors was inhibited and the progression of the disease was delayed [33–35].

## Conclusion

In this study, it was found that the collagen membrane could inhibit the degradation of chondrocytes and extracellular matrix by increasing the phosphorylation level of the p38MAPK protein, inhibiting the p38MAPK signaling pathway and decreasing the expression levels of MMP1 and MMP13 to promote the repair of articular cartilage defects. The interaction between P38MAPK and MMP cytokines is expected to be a new breakthrough in the treatment of osteoarthritis.
